# Versatility of MicroRNA Biogenesis

**DOI:** 10.1371/journal.pone.0019391

**Published:** 2011-05-10

**Authors:** Naama Volk, Noam Shomron

**Affiliations:** Department of Cell and Developmental Biology, Sackler Faculty of Medicine, Tel Aviv University, Tel Aviv, Israel; University of Pennsylvania, United States of America

## Abstract

MicroRNAs (miRNAs) are short single-stranded RNA molecules that regulate gene expression. MiRNAs originate from large primary (pri) and precursor (pre) transcripts that undergo various processing steps along their biogenesis pathway till they reach their mature and functional form. It is not clear, however, whether all miRNAs are processed similarly. Here we show that the ratio between pre-miRNA and mature miRNA forms varies between different miRNAs. Moreover, over-expression of several factors involved in miRNA biogenesis, including Exportin-5, Drosha, NF90a, NF45 and KSRP, displayed bidirectional effects on pre/mature miRNA ratios, suggesting their intricate biogenesis sensitivity. In an attempt to identify additional factors that might explain the versatility in miRNA biogenesis we have analyzed the contribution of two hnRNP family members, hnRNPH1 and hnRNPR. Knock-down or over-expression of these genes suggested that hnRNPR inhibits, whereas hnRNPH1 facilitates, miRNA processing. Overall, our results emphasize that miRNA biogenesis is versatile.

## Introduction

MicroRNAs (miRNAs) are short (∼22-nucleotide), endogenous, single-stranded RNA molecules that regulate gene expression by promoting RNA transcript degradation or translation inhibition of target mRNAs. MiRNA genes are transcribed in most cases by RNA polymerase II into primary miRNA transcripts (pri-miRNA) [1]. After transcription the primary microRNA (pri-miRNA) is cleaved by a complex called the microprocessor that contains DROSHA and DGCR8 (DiGeorge critical region 8) as well as additional proteins. DROSHA is an RNase III enzyme and DGCR8 is a protein that is essential for miRNA processing (also known as Pasha) [2]. The cleaved transcript, 70–110 nucleotide long, is referred to as precursor miRNA (pre-miRNA). After nuclear processing the pre-miRNA is exported to the cytoplasm by Exportin-5 in a complex with Ran-GTP61 [3]. Once the pre-miRNA is in the cytoplasm the RNase III protein Dicer cleaves off the loop of the pre-miRNA and generates a roughly 22-nucleotide miRNA duplex. The functional strand of the mature miRNA is loaded together with Argonaute (Ago2) proteins into RISC complex [4]. In principle, the miRNA duplex could give rise to two different mature miRNAs. However, in a similar manner to siRNA duplexes, only one strand is usually incorporated into RISC and guides the complex to target mRNAs.

An increasing number of proteins are being discovered as having key functions in miRNA maturation. For example, it has been shown that nuclear factors, NF-90 and NF-45 can inhibit pri-miRNA processing into pre-miRNA in the case of pri-let-7a and pri-miR-21 [5]. The KH-type splicing regulatory protein, KSRP, can serve as a component of both Drosha and Dicer complexes and regulates the biogenesis of a subset of miRNAs. KSRP binds with high affinity to the terminal loop of the target miRNA precursors and promotes their maturation [6]. It was suggested that the terminal loop is a pivotal structure where miRNA processing ‘activators’ (for example, KSRP) function in a coordinated way to convey proliferating, apoptotic or differentiating cues into changes of miRNA expression. Trabucchi et al. [6] suggested that KSRP is a key regulator of the processing of a sizeable subset of miRNA precursors (miR-1, miR-15, miR-16, miR-20a, miR-21, miR-26b, miR-27b, miR-106a, miR-125, miR-196, miR-206 and let-7) on the basis of its high-affinity binding to their terminal loop.

There are potentially additional factors involved in miRNA biogenesis with yet unknown roles. The DGCR8 complex contains 11 proteins that were shown to be associated with it [7]. These include hnRNPH1 and hnRNPR, both representing a subfamily of ubiquitously expressed heterogeneous nuclear ribonucleoproteins (hnRNPs). The hnRNPs are RNA binding proteins that form a complex with heterogeneous nuclear RNA [8]. These proteins are associated with pre-mRNAs in the nucleus and appear to influence pre-mRNA processing and mRNA stability and transport. While all of the hnRNPs are present in the nucleus, some seem to shuttle between the nucleus and the cytoplasm. The hnRNP proteins R and H1 have distinct nucleic acid binding properties and have three repeats of quasi-RRM domains that bind RNAs [9]. hnRNPR has been demonstrated to be involved in pre-mRNA splicing [10], [11] and to enhance transcription [12]. hnRNPH1 has been demonstrated to act as a splicing factor [13], [14].

The process of miRNA biogenesis appears to be applied to all miRNA species (excluding very few miRtrons; see [15]). However, it is not clear whether additional regulatory mechanisms facilitate or inhibit the production of miRNAs in various tissues and whether these act on all miRNAs expressed by a given cell type. If the rate of miRNA production in cells is similar to all miRNA species, it is expected that the ratio between pre-miRNA and mature forms would be roughly the same. In our study we analyzed miRNA processing in HeLa cells on both the pre-miRNA and mature miRNA levels and we tested the effect of over-expression of factors involved in miRNA processing on an array of miRNAs. Our results suggest that distinct miRNAs exhibit differential sensitivity to over-expression of tested factors. In addition we have identified two hnRNPs whose expression levels differentially alter the processing of miRNAs. The differential association of these hnRNPs with distinct pri-miRNA might explain their flexible processing. Altogether, our results emphasize the great processing versatility of distinct miRNAs during their biogenesis pathway.

## Materials and Methods

### Quantitative/real-time PCR (qPCR)

The cDNA for the quantification of pre-miRNAs and mature miRNAs was obtained from the same cells and RNA extracts (High Capacity cDNA Reverse Transcription Kit, Applied Biosystems, and miScript Reverse Transcription kit, QIAGEN, respectively). Levels of transcripts were measured for both pre and mature miRNAs by qPCR using the miScript SYBR Green PCR kit (QIAGEN). Mature miRNA qPCR detection was performed according to the manufactures' protocol. The pre-miRNA qPCR detection was performed according to the following protocol: 2× QuantiTect SYBR Green PCR Master Mix (12.5 µl); Forward/Reverse Primer (10 µM; 1 µl each); template cDNA and RNase free water (up to 25 µl). The cycle threshold (Ct) of each mature and pre-miRNA in the control (cells transfected with GFP) was subtracted from each sample that was trasfected with the different plasmids in order to receive the change in expression level (ΔΔct). Fold changes were obtained by using the formula 2∧-(ΔΔct).

### Cell growth and transfection

HeLa cells (ATCC) were grown in DMEM supplemented with 10% FCS, and penicillin/streptomycin (Sigma). Cells were transfected in 10 cm plates when the cells reached 75% confluence. Plasmid DNA (5 µg) was transfected together with 15 µl of lipofectamin in Opti-MEM (Invitrogen). In addition to the 5 µg plasmid DNA, 100 ng of GFP containing plasmid was added in order to measure transfection efficiency. Between 4–6 hours after the transfection the medium was replaced and cells were harvested 48 or 55 hr later. A fraction of the cells were analyzed by Western blot to detect the expression of the transfected constructs while the remainder was used for RNA extraction.

### Western Blot

Cells were washed with PBS and centrifuged. Lysis buffer (Tris 0.1 M+NaCl 150 nM pH = 7.4+1% NP40) and proteinase inhibitors were added to the pellet and the cells were incubated on ice for 10 min. After 10 min centrifugation, sample buffer was added to the supernatant and the pellet was boiled for 3 min then placed on ice. The samples were separated in a 10% polyacrylamide gel electrophoresis. For transfer to membrane a wet transfer was performed using an assembly of nitrocellulose membrane (Thermo Scientific/Pierce) and Whatmann paper. The transfer was performed at 100v, 280 mAmp for 1 hr and 40 min. After washes with PTW (PBS+20% Tween 20) membrane was blocked with 10% milk for 30 min. The first antibody was added (rabbit anti myc for Exportin-5, and mouse anti Flag for the rest of the proteins) to PTW containing 0.5% BSA for 1 hr. The second antibody (goat anti rabbit or donkey anti mouse) was added in 10% milk in PTW for 1 hr. Each step was separated by additional washes with PTW. Finally, after several washes with PTW (10 min each) ECL (Thermo Scientific/Pierce) was added for 5 min and the membrane was exposed to film.

### Gene expressing plasmids

Plasmids were obtained as follows: KSRP (pCMV-TAG2B-KSRP) from Dr. Roberto Gherzi, Gene Expression Regulation Lab, Experimental Oncology Istituto Nazionale Ricerca sul Cancro (IST), Italy; Exportin-5 (pk-myc-exp5) from Dr. Ian G. Macara, University of Virginia School of Medicine, USA; Drosha (pck-Drosha) from Dr. Narry Kim, Seoul University, South Korea; NF45 (pcDNA-Flag-NF45) and NF90a (pcDNA-Flag NF90a) from Dr. Shuji Sakamoto, Laboratory of Molecular Biology, Science Research Center, Kochi Medical School, Japan. Plasmids expressing hnRNPH1 and hnRNPR were constructed as follows. A Flag sequence was added to each reverse primer directed towards the hnRNPH1 and hnRNPR genes in order to generate a tagged protein. A Kozak sequence was added before the first ATG of each forward primer in order to increase protein expression. The amplified genes were inserted into the pcDNA3 plasmid backbone under the CMV promoter.

### RNA extraction

RNA was extracted from the cells using TRIzol reagent (Invitrogen) according to the manufacturers' instructions. Briefly, 1 ml of TRIzol was added to a 10 cm plate. The cells were transferred to a 1.5 ml tube with 200 µl chloroform and centrifuged for 10 min. After centrifugation, the clear aqueous phase was transferred to a new tube with 0.5 ml Isopropanol and centrifuged again. The RNA pellet was washed with 75% Ethanol. All RNA samples were then treated with DNase I (New England Biolabs) at 37° for 10 min following inactivation at 75° for 10 min. An additional cleaning step with phenol was usually required in order to remove any DNase or buffers left in the sample.

### Knock-down of hnRNPH1 and hnRNPR

Twenty-four hours prior to transfection cells were plated such that their confluence at the time of transfection will be 30–50%. Each 10 cm culture dish was transfected using Lipofectamine RNAiMAX Reagent (Invitrogen). RNAiMAX complexes were prepared by adding 75 pmol siRNA duplexes in 1 ml Opti-MEM and combined with 15 µl of Lipofectamine RNAiMAX that were diluted in 1 ml Opti-MEM. The siRNA duplex-Lipofectamine RNAiMAX complexes were added to each plate containing cells. Cells were incubated with the siRNA for 48 hr. Medium was changed after 4 hr. Subsequent siRNA transfection was performed 24 hr after the first one using the same protocol. For each transfection against hnRNPH1 and hnRNPR there was a control plate with scrambled RNA (the same amount as in the siRNA samples). The siRNA experiments were carried out three times each in triplicates.

### Immunoprecipitation (IP)

Ten cm culture dish with HeLa cells were transfected with 5 µg of pcDNA3-hnRNPH1-Flag, pcDNA3-hnRNPR-Flag or pcDNA3-Flag. After 48 hr cells were immunoprecipitated using EZview Red Anti-Flag M2 Beads (Sigma). At the end of the precipitation procedure, RNA was extracted using RNeasy Plus Mini Kit (Qiagen). Reverse transcription was obtained using High-Capacity cDNA Reverse Transcription kit (Applied Biosystems). All IP experiments were performed three times with triplicates in each experiment. Although the immuno-precipitation process is supposed to precipitate only the Flag tagged proteins, nonspecific binding occurs in all IP experiments.

## Results

### The ratio between pre- and mature miRNA levels varies for different miRNAs

During the miRNA biogenesis pathway pri-, pre- and mature miRNAs are formed. It was previously suggested that during the miRNA biogenesis pathway different isoforms may accumulate at all three stages of miRNA development. In order to asses whether this is true for miRNA biogenesis in HeLa cells, as well, we measured the accumulating levels of ten miRNAs using quantitative/real-time PCR (qPCR) (Table S1). We chose these particular ten miRNAs based on the specificity and ability of the qPCR method to detect them, and their relevance to cellular function. For most miRNAs chosen mature levels were higher than pre-miRNA levels indicating that at least in our chosen set miRNAs have much higher mature than pre- levels while for one – the opposite effect is observed. The fold difference between mature and pre-miRNAs varies greatly among the miRNAs tested suggesting that processing of miRNAs in HeLa cells fluctuate significantly between different miRNAs allowing accumulation at different maturation stages. We note that the primers designed for each pre-miRNA can amplify both the pre-miRNA and the pri-miRNA. Therefore, the levels of pre-miRNA obtained using this method represent both forms. Our result so far suggest that miRNA biogenesis in HeLa cells is versatile for different miRNAs. Given that all miRNAs are potentially exposed to the same cellular factors, such differential regulation could result from their sensitivity to the different miRNA biogenesis factors expressed in HeLa cells.

### Differential effect of miRNA biogenesis factors on miRNA processing

**Figure 1:**
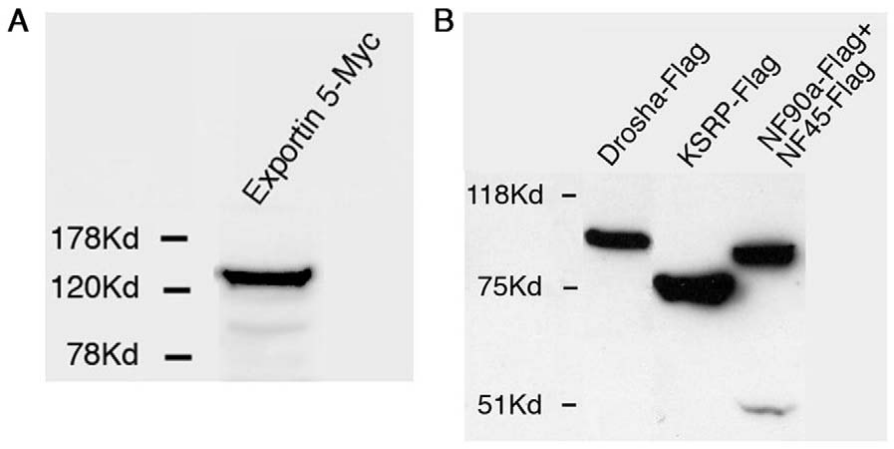
Expression levels of factors involved in miRNA biogenesis. Western blot analysis using (A) anti-myc antibody or (B) anti-Flag antibody of HeLa cells over-expressing either (A) Exportin-5, (B) Drosha, KSRP or NF-90a and NF-45.

In order to test whether any of the miRNA biogenesis factors are accountable for the observed differential levels of pre- and mature miRNA forms we over-expressed the following known miRNA biogenesis pathway genes: (i) Drosha, which cleaves the pri-miRNA into the pre-miRNA form of all but very few exceptional miRNAs; (ii) Nuclear factors NF90a and NF45, which inhibit the cleavage of pri-miRNA to pre-miRNA (shown for pri-let-7a and pri-miR-21) [5]; (iii) KSRP, which binds the terminal loop of the precursor and enhances its maturation (shown for miR-1, miR-15, miR-16, miR-20a, miR-21, miR-26b, miR-27b, miR-106a, miR-125, miR-196, miR-206 and let-7 on the basis of its high-affinity binding to their terminal loop) [6]; (iv) Exportin-5, which exports the pre-miRNA from the nucleus to the cytoplasm [3]. After allowing high levels of these proteins to accumulate in the cells (Fig. 1; 48 hr after transfection), RNA was extracted and the levels of pre- and mature miRNAs were quantified relative to control plasmid transfection.

**Figure 2:**
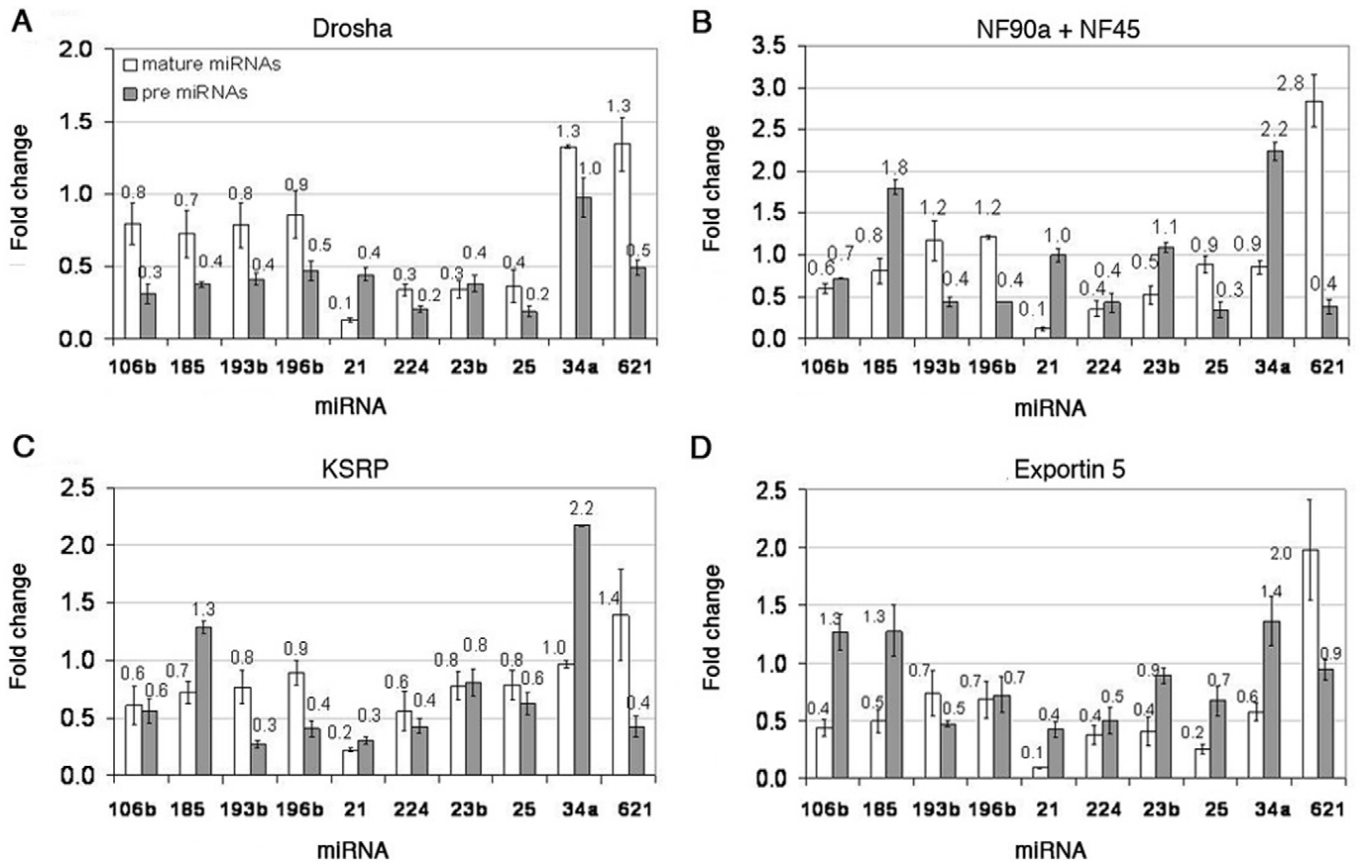
Fold change levels of mature and pre-miRNAs in transfected HeLa cells. Fold change levels of mature and pre-miRNAs in cells over-expressing (A) Drosha, (B) NF90a and NF45, (C) KSRP, or (D) Exportin-5.

Analysis of pre-miRNA and mature miRNA levels of over-expressing Drosha cells showed that in eight out of the ten miRNAs (miR-106b, miR-185, miR-193b, miR-196b, miR-21, miRNA-224, miR-23b, and miR-25), both the mature and pre-miRNA forms were decreased (Fig. 2A). In two out of the ten miRNAs (miR-34a and miR-621) the levels of the mature miRNA were elevated. In the case of miR-34a the pre-miRNA levels were unchanged whereas in the case of miR-621 the pre-miRNA levels decreased by two-fold. Increasing the levels of Drosha was expected to increase mature levels as a result of more rapid or efficient processing from pri- to pre-, however only two miRNAs (miR-34a and miR-621) showed this tendency.

Analysis of pre and mature miRNA levels of over-expressing NF90a and NF45 cells showed that in four out of the ten miRNAs (miR-185, miR-21, miR-23b and miR-34a), the pre-miRNA levels were elevated or unchanged whereas the mature levels were decreased (Fig. 2B). Three out of the ten miRNAs (miR-106b, miR-224 and miR-25) showed decreased levels of both mature and pre-miRNA forms. The remaining three miRNAs (miR-193b, miR-196b and miR-621) exhibited elevated mature levels and decreased pre-miRNA levels. Nuclear factors 90a and 45 (NF90a/45) were previously shown to inhibit the processing from pri- to pre-miRNA of pri-let-7a and pri-miR-21 [5]. Therefore, increasing their levels is expected to result in decreased mature levels and elevated/unchanged pre-miRNA levels. Indeed four of the ten miRNAs showed this tendency whereas the other six miRNAs showed other trends (Fig. 2B).

Analysis of pre- and mature miRNA levels of over-expressing KSRP cells, previously shown to promote the transition from pri to pre for several miRNAs, indicated that seven out of the ten miRNAs (miR-106b, miR-193b, miR-196b, miR-21, miR-224, miR-23b and miR-25) showed a variable decrease in the levels of both pre and mature forms (Fig. 2C). Two miRNAs (miR-185 and miR-34a) exhibited an increase of pre-miRNA levels, one – a slight decrease (miR-185) and the last – no change (miR-34a) in mature levels. One miRNA (miR-621) showed an increase in mature levels and a decrease in pre- levels. Interestingly, miR-106b, miR-185, miR-224, miR-25, miR-34a and miR-621 showed the same tendency as the over-expression of NF90a and NF45, suggesting that these factors affect them in a similar way. NF90 and KSRP are both translation regulatory RNA-binding proteins [16].

Over-expression of Exportin-5 led to significant shifts in the levels of pre- versus mature miRNAs. For six out of the ten miRNAs (miR-193b, miR-196b, miR-21, miR-224, miR-23b and miR-25), both the mature and the pre-miRNA forms were decreased (Fig. 2D). In three out of the ten miRNAs (miR-106b, miR-185 and miR-34a), the levels of the pre-miRNAs were elevated whereas the levels of the mature were decreased. One miRNA (miR-621) showed an increase in mature levels and no change in the pre levels. Over-expression of Exportin-5 was expected to facilitate the levels of mature miRNAs due to a more rapid or efficient export of the pre-miRNA from the nucleus to the cytoplasm. Interestingly, only one miRNA (miR-621) showed this tendency, namely an increase in mature levels whereas all the rest showed a decrease in mature levels.

In summary, our results suggest that even though miRNAs are potentially exposed to the same cellular factors their biogenesis is individually and intricately regulated. This also suggests that the binding of miRNA biogenesis factors to the assembling miRNA transcript might be a regulatory step controlling the levels of the different miRNA forms.

### hnRNPH1 and hnRNPR bind the pri-miRNA transcripts

**Figure 3:**
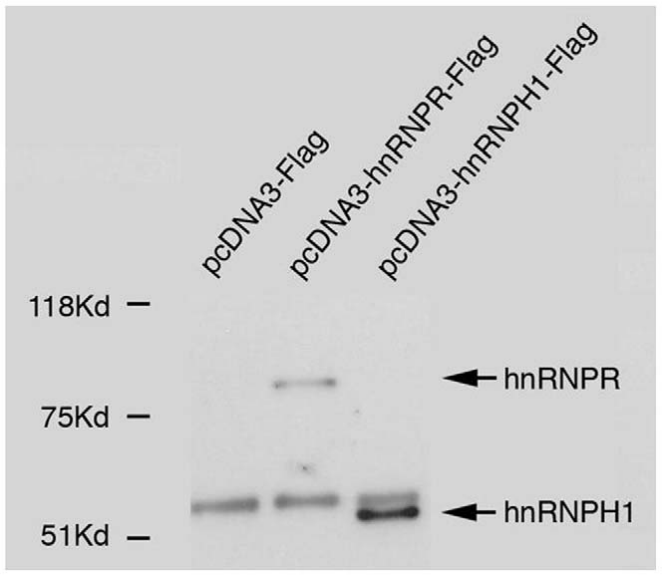
Western blot analysis performed on immunoprecipitated hnRNPR and hnRNPH1. Western blot analysis with anti Flag antibody performed on samples immunoprecipitated with anti-Flag M2 beads. The band that appears in all three samples around 50 kDa represents the heavy chain of the IgG.

We have shown that miRNA biogenesis factors, known to bind miRNA transcripts during their maturation process, affect miRNA levels distinctively. In an attempt to identify additional genes that would explain the variability in the processing of the ten miRNAs analyzed in our study, we decided to focus on the function of two candidate RNA binding proteins (RNPs); hnRNPH1 and hnRNPR. These RNPs are part of 11 proteins that have been shown to be associated with the DGCR8 complex and in addition they are known to be involved in RNA processing [7]. To address whether these RNPs exert their affect directly or via mediators we performed immunoprecipitation experiments asking whether pre-miRNAs are bound to hnRNPH1 or hnRNPR proteins. Following successful immunoprecipitation of these proteins (note that negative control Flag-only peptides were not precipitated; Fig. 3), RNA was extracted from the precipitates and the levels of pri-miRNAs were analyzed using qPCR. We interpreted the amount of each pre-miRNA as the affinity by which it was bound to its precipitated protein. The miRNAs were divided into three groups – low, medium and high association with the hnRNPs – representing different degrees of affinity, according to the amount of pri-miRNA versus the negative control vector (Table 1) (see Fig. S1 for an additional negative control). We further segregated the detected pri-miRNAs in each sample into three groups. In the first group the fold changes of the pri-miRNA levels relative to control were low (x1–3). In the second group the fold changes of the pri-miRNA levels relative to control were intermediate (x3–6). While in the third group the fold changes of the pri-miRNA levels relative to control were high (x6 and up) (see Table S2). Interestingly, the pri miRNAs showed different affinities to the two proteins. For example, pri-miRNA-34a showed a high affinity towards hnRNPH1 but only a medium affinity to hnRNPR whereas pri-miRNA-21 had a low affinity towards hnRNPH1 and a medium affinity towards hnRNPR. To exclude the possibility that the high affinity of the pri-miRNAs was correlated with the total amount of pri/pre-miRNA in the cell, the levels of the pre (and pri) miRNA in untreated cells were measured (Fig. S2). The results clearly demonstrated that there is no correlation between the amounts of a given pre/pri miRNA in the cell and the ability of the RNPs to precipitate it. For example, pri-miR-621 exhibits the highest levels of pre-miRNAs in the cell but it only shows a medium affinity to both RNPs. Pri-miR-34a is one of the least abundant miRNAs (Fig. S2) however it exhibits a high affinity to the hnRNPH1 protein. It is thus most likely that the co-immunoprecipitation experiments correspond to a genuine affinity of the pri-miRNA to the hnRNPs. These results demonstrate that both hnRNPs form protein-RNA complexes with distinct pri-miRNAs, and thus hnRNPH1 and hnRNPR are directly involved in the processing of miRNAs.

**Table 1:** Degrees of binding affinity of pri-miRNAs to hnRNPH1 and hnRNPR proteins following immunoprecipitation.

Binding of protein to pri-miRNAs	hnRNPH1			hnRNPR		
miR\Binding	Low	Medium	High	Low	Medium	High
pri-miR-34a			+		+	
pri-miR-621		+			+	
pri-miR-21	+				+	
pri-miR-185			+	+		
pri-miR-193b			+			+
pri-miR-106b		+		+		
pri-miR-196b			+			+
pri-miR-224		+		+		
pri-miR-23b		+		+		
pri-miR-25		+		+		

The extent of binding of hnRNPR and hnRNPH1 to the 10 selected miRNAs.

### Reduction of hnRNPH1 and hnRNPR levels changes miRNA processing

**Figure 4:**
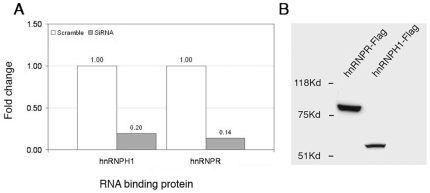
Fold change and expression of hnRNPR and hnRNPH1 following their knock-down or over expression. (A) Fold change levels of hnRNP mRNAs in cells following knock-down with siRNA for 48 h. (B) Western blot analysis using anti-Flag antibody of HeLa cells over-expressing hnRNPH1 and hnRNPR.

**Figure 5:**
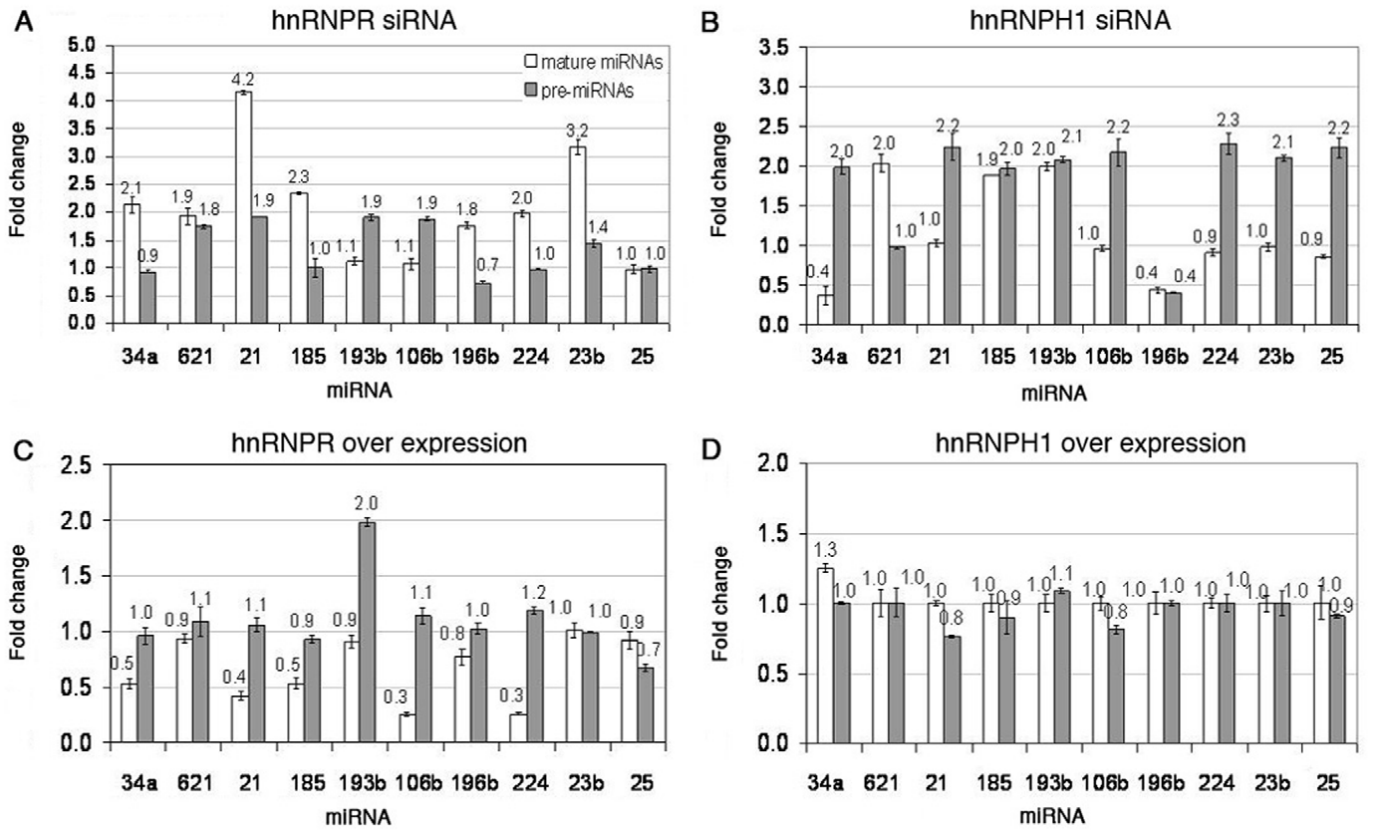
Fold change levels of mature and pre-miRNAs in HeLa cells in which hnRNPs were knocked down or over expressed. Fold change levels of mature versus pre-miRNAs in cells following knock-down of (A) hnRNPR or (B) hnRNPH1 by siRNA for 48 h and following over-expression of (C) hnRNPR or (D) hnRNPH1.

We have shown using immunoprecipitation experiments that hnRNPR and hnRNPH1 bind pri-miRNAs. In order to see whether these proteins exhibit a direct effect on miRNA biogenesis, we analyzed miRNA processing following their over- and under-expression. hnRNPH1 and hnRNPR mRNA levels were significantly reduced in HeLa cells by 5 and 7 fold, respectively (Fig. 4A). Analysis of pre and mature levels of cells with reduced hnRNPR amounts exhibited that all mature miRNAs levels have increased (miR-34a, miR-621, miR-21, miR-185, miR-196b, miR-224 and miR-23b) or have remained unchanged (miR-193b, miR-106b and miR-25) (Fig. 5A). This finding suggests that hnRNPR may have an inhibitory role in the miRNA biogenesis pathway and therefore reducing its levels resulted in increasing levels of many of the miRNAs. Pre-miRNAs levels mostly remained unchanged or have slightly increased. The phenomenon was robust and persisted at various gene knock-out levels, cell confluence and time periods (data not shown). Analysis of pre and mature miRNA levels in cells with reduced hnRNPH1 mRNA amounts exhibited an increase of pre-miRNA levels in eight out of the ten miRNAs (miR-34a, miR-21, miR-185, miR-193b, miR-106b, miR-224, miR-23b and miR-25) (Fig. 5B). This finding suggests that hnRNPH1 may have a positive role in the miRNA biogenesis pathway and therefore reducing its levels might result in increased levels of most pre-miRNAs. One pre-miRNA level was decreased (miR-196b) while another was unchanged (miRNA-621).

### Over-expression of hnRNPH1 and hnRNPR changes miRNA processing

Both hnRNPH1 and hnRNPR showed a dramatic, yet opposite, effect on miRNA biogenesis of the ten miRNAs examined. In order to test whether this phenomenon is reversible we over-expressed these genes (Fig. 4B) and looked at pre- and mature miRNA levels. Over-expression of hnRNPR led to a decrease in the levels of mature miRNAs (miR-34a, miR-21, miR-185, miR-106b, miR-196b and miR-224) or did not change their levels (miR-621, miR-193b, miR-25 and miR-23b) (Fig. 5C). The pre-miRNA levels have remained the same for nine out of the ten miRNAs. Pre-miRNA-193b exhibited increased levels by two fold. These results support the possible inhibitory role of hnRNPR on miRNA processing. Analysis of pre- and mature miRNA levels in over-expressing hnRNPH1 cells showed that all pre- and mature miRNAs have remained unchanged (Fig. 5D).

## Discussion

In our study we showed that different types of miRNAs are processed in different manners in HeLa cells. Moreover, the sensitivity of each miRNA to over-expression of general factors involved in miRNA biogenesis differs significantly. Importantly, we have identified two hnRNP proteins, hnRNPH1 and hnRNPR, that are associated with pri-miRNAs and influence their processing in HeLa cells. Furthermore, we showed that hnRNPR exhibits an inhibitory role in miRNA processing while hnRNPH1 functions to facilitate miRNA processing. Although these proteins were described to be part of the DGCR8 complex we have demonstrated, for the first time, their involvement in miRNA processing. The sensitivity of the miRNAs to depletion or over-expression of these genes differs between the different types of miRNAs.

### The difference in the levels of mature and pre-miRNA forms

Our results suggest that the ratio between mature and pre-miRNA forms differ between distinct miRNA types. The data obtained in our experiments implied that there is variability in the rate of processing between different miRNAs in HeLa cells. We chose ten miRNAs based on the specificity and accuracy of the qPCR method to detect them, and their relevance to cellular function, for further analysis. The group of miRNAs we chose to further analyze includes miR-21 which acts as an oncomir in prostate and other cancers (for example see [17] and the miR-106b-25 miRNA cluster which has a role in hepatocellular carcinoma [18]). The qPCR analysis clearly demonstrated that in nine out of the ten miRNAs analyzed the mature miRNA levels were significantly higher than the pre-miRNA levels. This observation could be interpreted by two alternative explanations: (i) the half-lives of these mature miRNAs are high and therefore they remain stable for a long period of time; (ii) the processing rate of these miRNAs is high, meaning that the transition rate from pri/pre forms to mature miRNA is rapid. One miRNA (miR-621) showed an opposite tendency. The pre levels were high while its mature levels were relatively low. This could be explained by the fact that: (i) the pre-miRNA is very stable; (ii) the processing rate of this miRNA from pri/pre form to mature miRNA is slow which means that most of the miRNA is left in its pri/pre form. In both cases we do not have information about the rate of transcription for these miRNAs which could be an additional factor affecting the changes observed in the ratio between mature and pre-miRNA for the different miRNA types. These results are consistent with the hypothesis that different miRNA types are processed at a differential rate.

### The effect of over-expression of factors involved in miRNA processing on the levels of mature and pre-miRNAs

To asses whether the different processing rates of the miRNAs occur due to their differential sensitivity to the levels of known biogenesis factors, we over-expressed several of them in HeLa cells and analyzed the levels of pre- and mature miRNAs. The factors tested included: Exportin-5, Drosha, NF90a/NF45 and KSRP. All the genes were expressed at high levels (as indicated by Western blot analysis, except NF45 which exhibited a low expression level, at least 5 fold). Although it has been shown that the maximum effect on miRNA processing occurs when both NF90 and NF45 are transfected together, NF90 exhibited an effect on miRNA processing when over-expressed alone [5], therefore the relatively low levels of NF45 were not expected to affect the results in a quantitative manner.

### Exportin-5 over-expression

Over-expression of Exportin-5 was expected to increase the levels of mature miRNAs [3] but instead in nine out of ten miRNAs the levels of the mature miRNA were decreased. Only miR-621 showed an increase in mature levels as expected. Possible explanations for the fact that most miRNAs did not show an increase in mature levels are: (i) that the pre-miRNA levels were low at the beginning of the experiment, over-expression of Exportin-5 would not change mature levels because there would be no excess pre-miRNA to export from the nucleus; (ii) it was shown that Exportin-5 contributes to the stability of the pre-miRNA. It might be possible that over-expression of Exportin-5 stabilized the pre-miRNA forms to such degree that it prevents further processing and therefore lead to a decrease in the mature levels [3]; (iii) Exportin-5 levels in the cells are saturated; (iv) an excess of Exportin-5 could associate with other factors which lead to their exclusion from the nucleus and to reduced miRNA biogenesis activity. For example, binding of an excess of Exportin-5 to GTP could decrease GTP levels in the nucleus and therefore slow the export process instead of increasing it. The only miRNA that showed elevated mature levels in response to over-expression of Exportin-5 was miR-621. Possible explanations for this observation are: (i) miR-621 exhibits very high pre-miRNA levels and therefore elevated Exportin-5 indeed facilitated its export to the cytoplasm promoting its maturation; (ii) this miRNA exhibits unique affinity to Exportin-5 which takes first priority in export over the other miRNAs; (iii) the half-life of the mature miRNA is longer for miR-621.

### Drosha, NF90a, NF45 and KSRP over-expression

Over-expression of Drosha, an enzyme that is involved in the pri to pre-miRNA processing, increased mature levels of two out of the ten miRNAs. This was in contrast to the expected effect of increased mature miRNA levels. Only miR-34a and miR-621 showed subtle elevated mature levels. Interestingly, pre- levels of most miRNAs decreased by two-fold. Possible explanations for these observations are: (i) Drosha works with DGCR8 which was not over-expressed in this experiment. If DGCR8 is a limiting factor then Drosha alone would not have a strong impact on miRNA processing; (ii) an excess of Drosha could deplete other factors that usually work in concert with the complex. This could result in accumulation of pre-miRNAs. It was shown that the complex of Nuclear Factor 90 (NF90) and NF45 proteins functions as a negative regulator in miRNA biogenesis in the case of pri-let-7a and pri-miR-21 [5]. For these miRNAs, pri-miRNA processing into pre-miRNA was inhibited by over-expression of the NF90 and NF45 proteins. In our experiments, over-expression of NF90a and NF45 promoted a decrease in mature levels in four out of the ten miRNAs. One miRNA, miR-21, was also affected in the Sakamoto study [5] and shows consistency with their observation. These results suggest that NF90a and NF45 might influence the processing of a subgroup of miRNAs. MiR-621 showed an opposite tendency relative to the rest of the miRNAs in response to over-expression of NF90a and NF45. It is possible that this miRNA utilizes a different processing pathway. KSRP facilitates the transition between pri and pre forms for some miRNAs [6]. Therefore over-expression of KSRP is expected to elevate mature miRNA levels. However, only miR-621 showed this tendency. This could be due to the fact that KSRP works on some but not all miRNAs. In addition, an increase in the transition between pri and pre-miRNA levels may not result in an automatic increase in the mature miRNA levels due to additional factors involved along the pathway.

### The involvement of hnRNPR and hnRNPH1 in miRNA biogenesis

Although it was shown that hnRNPH1 and hnRNPR are part of the DGCR8 complex, their direct involvement in the miRNA processing pathway has not been investigated.

In order to asses whether the effect of hnRNPR is direct and depends on the ability of hnRNPR to be in association with a given pri-miRNA, we performed immunoprecipitation experiments of protein-RNA complexes. The results show that the binding of hnRNPR to the pri-miRNAs was specific; however the affinity of the binding varied between the miRNAs. In certain cases there is a correlation between the ability of the protein to bind a certain pri-miRNA and its ability to affect miRNA processing following knock-down by siRNA (miR-106b, miR-25, miR-196b, miR-21, miR-621 and miR-34a), suggesting that hnRNPR acts in a way that depends on its affinity to the pri-miRNA (Fig. S3). In addition, some miRNAs that were elevated following knock-down of hnRNPR were decreased following its over-expression (miR-25, miR-196b, miR-21, miR-621 and miR-34a). However, additional factors might influence its inhibitory activity explaining cases in which there was no correlation between the binding affinity and the activity of this protein.

In order to test for a more direct involvement of these proteins in miRNA biogenesis, we have knocked-down their levels in HeLa cells. The knock-down at the mRNA level was highly efficient and decreased their levels significantly (Fig. 4).

### hnRNPR exhibits an inhibitory effect on miRNA maturation

Reducing hnRNPR levels led to an increase in mature miRNA levels of seven out of ten miRNAs examined. The pre levels mostly remained unchanged or were slightly increased. The increase in mature levels suggests that hnRNPR has an inhibitory role in miRNA processing and therefore knocking-down its levels led to an increase in miRNA processing. Possible explanations for hnRNPR inhibitory activity might be its binding to the pri-miRNA and inhibiting its further processing by Drosha or causing its destabilization. Consistent with the idea that hnRNPR plays an inhibitory role; over-expression of this protein in HeLa cells caused a decrease in most of the mature miRNAs. However, there was variability in the response of the miRNAs to the over-expression of hnRNPR. Significantly, most miRNAs that responded positively to the siRNA of hnRNPR decreased their levels in response to over-expression of hnRNR, suggesting that hnRNPR is necessary and sufficient for inhibiting miRNA processing in the tested group of miRNAs. Two miRNA (miR-25 and miR-193b) did not respond to either of the transfections, suggesting an independent or different processing pathway that may not involve hnRNPR.

### hnRNPH1 might promote miRNA processing

Knock-down of hnRNPH1 caused variable effects. The pre-miRNA levels in most miRNAs showed an increase suggesting a positive role for hnRNPH1 in miRNA processing. Two miRNAs, miR-196b and miR-621 showed decrease or unchanged pre levels respectively. Blocking of pre-miRNA processing and an increase in the pre-miRNA levels is expected to result in decreased levels of the mature miRNAs. However, the mature miRNA levels showed variable tendencies. It is possible that each miRNA has its own half-life and in the event that the miRNA are highly stable, they will not degrade easily, and therefore their levels will remain unchanged. In addition, blocking hnRNPH1 may result in slowing of the miRNA processing but it might not completely arrest it, therefore some miRNAs will still be processed. Over-expression of hnRNPH1 did not show any significant changes in miRNA levels. This might be explained by the fact that hnRNPH1 exists in saturated levels in the cell and therefore over-expression does not affect the miRNA processing. The binding experiment with hnRNPH1 showed that there is a correlation between the binding of hnRNPH1 to a certain pri-miRNA and the contribution of this protein to miRNA processing, as implicated from the siRNA experiment. MiRNAs that showed this tendency are miR-106b, miR-25, miR-224, miR-196b, miR-21, miR-23b and miR-34a (Fig. S4). These results support the hypotheses that hnRNPH1 affects miRNA processing through binding to pri-miRNAs.

Taken together these results support our hypothesis that hnRNPR and hnRNPH1 exhibit a direct role in the processing of pri-miRNAs. In addition we show for the first time that hnRNPR exhibits an inhibitory activity whereas hnRNPH1 exhibits a facilitating role during miRNA processing. These activities are presumably mediated by their binding to the pri-miRNAs. In addition our results indicate that there is a great versatility and variability in the processing of different miRNAs in HeLa cells. This variability could stem from the involvement of certain factors like hnRNPR and hnRNPH1 which might exhibit different affinities to different pri or pre-miRNAs. The advantage of regulation of specific miRNAs through their processing provides the cell means to temporarily regulate the activity of the miRNAs so that it would not reduce the levels of important targets prematurely. The accumulation of pri/pre-miRNAs within the cells enables the cell to respond rapidly when the miRNA is needed. Therefore factors such as hnRNPH1 and hnRNPR might be important to ensure correct pre- to mature miRNA ratio which enables prompt response of the cell to different conditions.

In summary, we have shown for the first time that miRNAs undergo differential processing within HeLa cells and responded differently to different factors involved in miRNA biogenesis. Two additional factors, hnRNPH1 and hnRNPR, were shown to directly influence miRNA processing, either by inhibiting (hnRNPR) or promoting (hnRNPH1) miRNA maturation of certain miRNAs.

## Supporting Information

Figure S1 **GAPDH levels measured in cells expressing pcDNA3-hnRNPH1-Flag, pcDNA3-hnRNPR-Flag or pcDNA3-Flag and immunoprecipitated using anti-Flag M2 beads.**
(TIF)

Figure S2 **Levels of pre (and pri) miRNAs in untreated HeLa cells.**
(TIF)

Figure S3 **Fold change following siRNA of hnRNPR.** Fold change of pri-miRNAs in HeLa cells following siRNA against hnRNPR and in correlation to the binding affinity of hnRNPR.
(TIF)

Figure S4 **Fold change following siRNA of hnRNPH1.** Fold change of pri-miRNAs in HeLa cells following siRNA against hnRNPH1 and in correlation to the binding affinity of hnRNPH1.
(TIF)

Table S1 The levels of mature and pre-miRNAs analyzed using Quantitative/Real Time PCR. The pink colored miRNAs represent miRNAs that exhibit high levels of mature miRNA and low levels of pre-miRNAs. The blue colored miRNA represents the miRNA that exhibit low levels of mature miRNA and high levels of pre-miRNA.
(DOC)

Table S2 Fold change of pri-miRNAs in HeLa cells following immunoprecipitation of either hnRNPH1 or hnRNPR. Fold change is normalized to an empty pcDNA3-Flag vector.
(DOC)
