# Supplementary figures and images for: Versatility of MicroRNA Biogenesis
Source: PLoS One. 2011 May 10;6(5):e19391. doi: 10.1371/journal.pone.0019391 (PMC3091858; doi:10.1371/journal.pone.0019391)

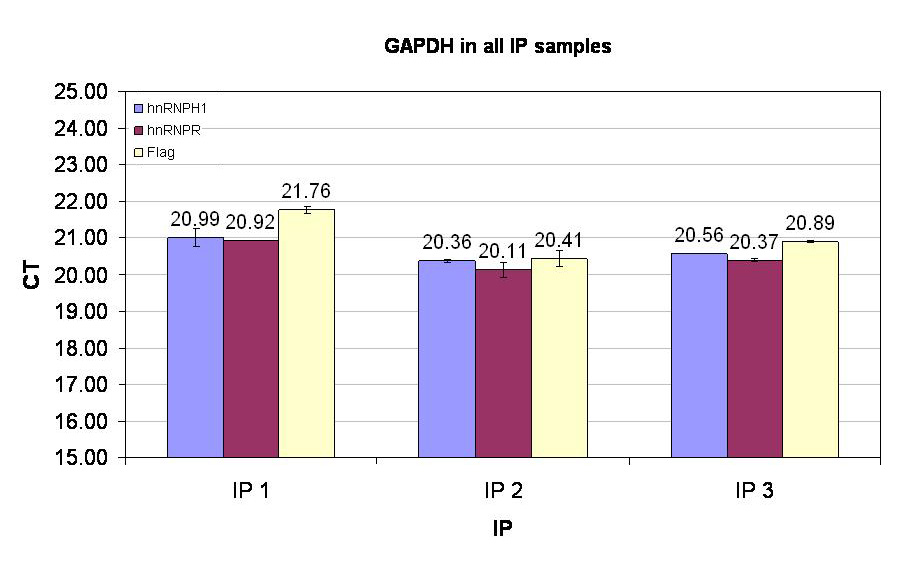

Supplement: Figure S1 — GAPDH levels measured in cells expressing pcDNA3-hnRNPH1-Flag, pcDNA3-hnRNPR-Flag or pcDNA3-Flag and immunoprecipitated using anti-Flag M2 beads. (TIF) [file pone.0019391.s001.tif]

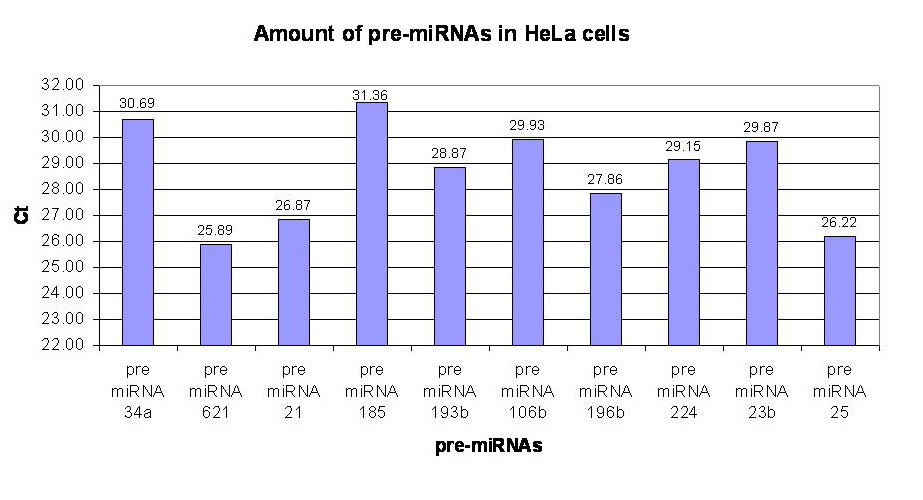

Supplement: Figure S2 — Levels of pre (and pri) miRNAs in untreated HeLa cells. (TIF) [file pone.0019391.s002.tif]

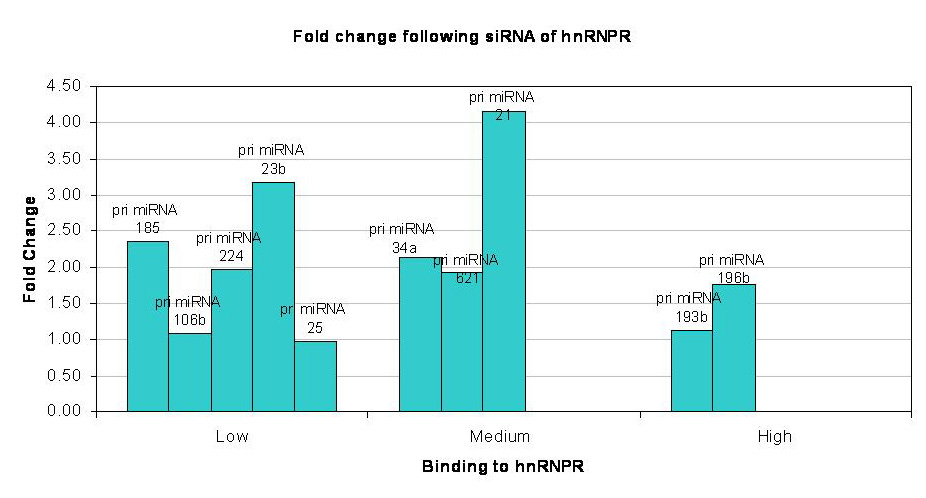

Supplement: Figure S3 — Fold change following siRNA of hnRNPR. Fold change of pri-miRNAs in HeLa cells following siRNA against hnRNPR and in correlation to the binding affinity of hnRNPR. (TIF) [file pone.0019391.s003.tif]

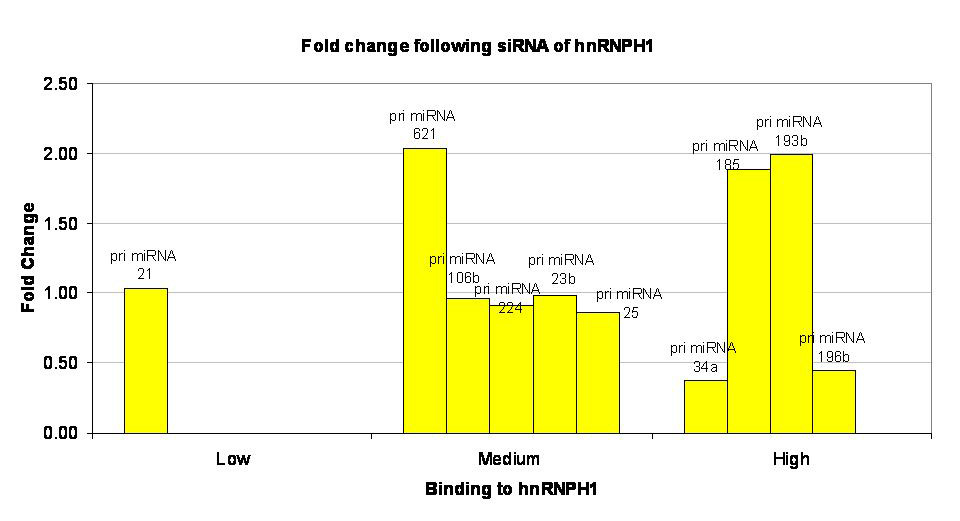

Supplement: Figure S4 — Fold change following siRNA of hnRNPH1. Fold change of pri-miRNAs in HeLa cells following siRNA against hnRNPH1 and in correlation to the binding affinity of hnRNPH1. (TIF) [file pone.0019391.s004.tif]
