# Supplementary material for: Versatility of MicroRNA Biogenesis
Source: PLoS One. 2011 May 10;6(5):e19391. doi: 10.1371/journal.pone.0019391 (PMC3091858; doi:10.1371/journal.pone.0019391)
Supplement: Table S1 — The levels of mature and pre-miRNAs analyzed using Quantitative/Real Time PCR. The pink colored miRNAs represent miRNAs that exhibit high levels of mature miRNA and low levels of pre-miRNAs. The blue colored miRNA represents the miRNA that exhibit low levels of mature miRNA and high levels of pre-miRNA. (DOC) [file pone.0019391.s005.doc]

**Table S1**

|  | **Ct**  **mature miR** | **Ct**  **pre-miR** | **Fold Change (mature-pre)** |
| --- | --- | --- | --- |
| **miR-106b** | 21.43 | 28.70 | 154 |
| **miR-185** | 22.93 | 32.16 | 599 |
| **miR-193b** | 19.03 | 27.93 | 477 |
| **miR-196b** | 20.60 | 26.70 | 68 |
| **miR-21** | 16.02 | 27.42 | 2711 |
| **miR-224** | 20.21 | 28.13 | 242 |
| **miR-23b** | 15.98 | 28.53 | 5960 |
| **miR-25** | 19.49 | 25.60 | 68 |
| **miR-34a** | 24.94 | 31.73 | 110 |
| **miR-621** | 28.63 | 24.67 | 0.06 |
