# Supplementary material for: Versatility of MicroRNA Biogenesis
Source: PLoS One. 2011 May 10;6(5):e19391. doi: 10.1371/journal.pone.0019391 (PMC3091858; doi:10.1371/journal.pone.0019391)
Supplement: Table S2 — Fold change of pri-miRNAs in HeLa cells following immunoprecipitation of either hnRNPH1 or hnRNPR. Fold change is normalized to an empty pcDNA3-Flag vector. (DOC) [file pone.0019391.s006.doc]

**Table S2**

| hnRNPR IP 3 | hnRNPR IP 2 | hnRNPR IP 1 | hnRNPH1 IP 3 | hnRNPH1 IP 2 | hnRNPH1 IP 1 | Target Name |
| --- | --- | --- | --- | --- | --- | --- |
| 3.05 | 5.47 | 2.76 | 3.43 | 3.86 | 3.14 | miR-621 |
| 2.83 | 2.50 | 1.62 | 8.07 | 10.33 | 4.42 | miR-185 |
| 5.09 | 5.80 | 3.59 | 2.83 | 2.37 | 1.54 | miR-21 |
| 2.21 | 1.58 | 1.14 | 4.31 | 4.52 | 3.06 | miR-25 |
| 1.58 | 1.45 | 1.70 | 3.74 | 4.45 | 4.32 | miR-106b |
| 10.42 | 9.69 | 5.53 | 21.07 | 26.96 | 11.95 | miR-193b |
| 3.63 | 7.71 | 3.80 | 14.72 | 17.86 | 10.77 | miR-196b |
| 2.16 | 2.92 | 1.82 | 5.75 | 6.60 | 4.21 | miR-224 |
| 3.66 | 2.19 | 1.69 | 3.46 | 4.09 | 2.50 | miR-23b |
| 3.28 | 3.24 | 2.14 | 13.07 | 13.86 | 6.35 | miR-34a |
